# A Loop-Mediated Isothermal Amplification Assay for the Rapid Detection of *Didymella segeticola* Causing Tea Leaf Spot

**DOI:** 10.3390/jof10070467

**Published:** 2024-07-03

**Authors:** Yiyi Tu, Yuchun Wang, Hong Jiang, Hengze Ren, Xinchao Wang, Wuyun Lv

**Affiliations:** 1 College of Tea Science and Tea Culture, Zhejiang A&F University, Hangzhou 311300, China; 2022614022068@stu.zafu.edu.cn (Y.T.); ycwang0201@zafu.edu.cn (Y.W.); hulkww@zafu.edu.cn (H.J.); renhengze@zafu.edu.cn (H.R.); 2 Tea Research Institute, Chinese Academy of Agricultural Sciences/National Center for Tea Improvement/Key Laboratory of Biology, Genetics and breeding of Special Economic Animals and Plants, Ministry of Agriculture and Rural Affairs, Hangzhou 310008, China

**Keywords:** tea plant, *Didymella segeticola*, diagnosis, specificity, efficiency

## Abstract

Tea leaf spot caused by *Didymella segeticola* is an important disease that threatens the healthy growth of tea plants (*Camellia sinensis*) and results in reductions in the productivity and quality of tea leaves. Early diagnosis of the disease is particularly important for managing the infection. Loop-mediated isothermal amplification (LAMP) assay is an efficient diagnostic technique with the advantages of simplicity, specificity, and sensitivity. In this study, we developed a rapid, visual, and high-sensitivity LAMP assay for *D. segeticola* detection based on sequence-characterized amplified regions. Two pairs of amplification primers (external primers F3 and B3 and internal primers FIP and BIP) were designed based on a specific sequence in *D. segeticola* (NCBI accession number: OR987684). Compared to common pathogens of other genera in tea plants and other species in the *Didymella* genus (*Didymella coffeae-arabicae*, *Didymella pomorum*, and *Didymella sinensis*), the LAMP method is specific for detecting the species *D. segeticola*. The assay was able to detect *D. segeticola* at a minimal concentration of 1 fg/μL genomic DNA at an optimal reaction temperature of 65 °C for 60 min. When healthy leaves were inoculated with *D. segeticola* in the laboratory, the LAMP method successfully detected *D. segeticola* in diseased tea leaves at 72 h post inoculation. The LAMP assays were negative when the DNA samples were extracted from healthy leaves. Leaf tissues with necrotic lesions from 18 germplasms of tea plants tested positive for the pathogen by the LAMP assay. In summary, this study established a specific, sensitive, and simple LAMP method to detect *D. segeticola*, which provides reliable technical support for estimating disease prevalence and facilitates sustainable management of tea leaf spot.

## 1. Introduction

Tea plant [*Camellia sinensis* (L.) O. Kuntze] is a perennial woody species that is widely cultivated in tropical and subtropical areas such as China, India, Sri Lanka, Kenya, and Japan [1,2,3]. Tea plants often suffer from various diseases caused by pathogenic fungi, which threaten global tea production [2,4,5,6,7]. Zhao et al. (2018) reported that tea leaf spot disease caused by *Didymella segeticola* (Q. Chen) Q. Chen, Crous & L. Cai severely affects the quality and yield of tea leaves [8]. *D. segeticola* invades young leaves or mature leaves, resulting in small needle-like brown spots surrounded by a yellow halo at the early stage of infection; the lesions gradually expand and fuse into irregular spots, and the center becomes grayish-brown to grayish-white; diseased leaves are deformed and twisted, easily fall off, and grow slowly in the later stage of infection [8,9]. Leaf spots have become common foliar diseases of tea plants in recent years, resulting in a decrease in tea productivity and affecting quality and flavor by changing the composition and content of key secondary metabolites [8,9,10]. Early detection and subsequent development of strategies to manage this disease are therefore urgently needed and crucially important [11].

Traditionally, disease diagnosis involving the isolation, identification, and characterization of pathogens is strenuous, time-consuming, and microscopy-dependent [12,13]. Recently, molecular diagnostic methods based on some conserved housekeeping sequences, such as ribosomal RNA-encoding DNA sequences, the glyceraldehyde-3-phosphate dehydrogenase-encoding gene, and cytoskeletal protein-encoding genes, have been used for the identification of pathogens that are difficult to distinguish morphologically [11,14,15,16,17]. On this basis, polymerase chain reaction (PCR) has been routinely used for disease diagnosis and detection of a large range of plant pathogenic fungi [18]. Real-time PCR (quantitative PCR, qPCR), reverse transcription PCR, nested PCR, and droplet digital PCR have further been developed as new DNA-based molecular diagnostic tools [18,19,20,21,22]. In addition, terminal restriction fragment length polymorphism (T-RFLP), immunological methods, DNA microarray, denaturing gel gradient electrophoresis, and next-generation sequencing platforms are also applied to identifying pathogens [18,23,24,25]. However, these technologies have common limitations, such as complex experimental designs, costly consumables, consumed time, trained manpower, and specific apparatuses, even though they can specifically identify pathogens [11]. Thus, many isothermal nucleic acid amplification techniques have been developed and applied for disease diagnosis because they do not require specific apparatuses, such as thermocyclers, electrophoresis devices, and UV light [22]. These techniques mainly include cross-priming amplification (CPA), helicase-dependent amplification (HDA), loop-mediated isothermal amplification (LAMP), nucleic acid sequence-based amplification (NASBA), polymerase spiral reaction (PSR), strand-displacement amplification (SDA), rolling circle amplification (RCA), and recombinase polymerase amplification (RPA) [22,26,27,28,29,30,31,32].

LAMP is an efficient and powerful technique that can be used for the molecular detection of plant pathogenic fungi in disease diagnosis [33,34,35,36,37]. Conventionally, at least four specific primers, including two external primers (F3 and B3) and two internal primers (forward inner primer, FIP, and backward inner primer, BIP), are needed to recognize six different regions in the target DNA with a strand-displacing DNA polymerase under isothermal conditions [22,30]. The LAMP reaction was carried out at 60–65 °C within a short reaction time (<1 h), eliminating the dependence on a thermocycler [38]. With low instrument requirements, it can be performed in a water bath or heating block [30,39]. LAMP is thus simple, rapid, specific, highly sensitive, and efficient [34]. Furthermore, the LAMP product can be easily visualized by adding magnesium titration with hydroxy naphthol blue, a pH-sensitive dye, and fluorescent compounds such as SYBR Green I, calcein, EvaGreen, SYTO, and berberine [40,41,42,43]. With these advantages, the LAMP method has been widely used for the detection of many plant-pathogenic fungi [44,45,46,47,48,49]. For example, the LAMP assay is a useful and convenient method for directly detecting *Colletotrichum truncatum* in diseased soybean tissues [49]. A set of four primers exhibiting high species specificity and sensitivity that target the *Rpb1* (encoding the large subunit of RNA polymerase II) sequence of *C. truncatum* was selected for further study. After amplification at 62 °C for 70 min, the LAMP products turned yellow-green in the presence of *C. truncatum* when SYBR Green I was added. The sensitivity of the LAMP assay was determined, and the results showed that the minimum concentration of *C. truncatum* DNA detected in the assay was 100 pg μL^−1^ [49]. For pathogenic fungi isolated from tea plants, the LAMP assay has been used for the rapid and precise detection of *C. siamense* and *Exobasidium vexans* [50,51]. However, a LAMP method has not yet been developed for the detection of *D. segeticola*, which is the causal agent of tea leaf spot.

In this study, we developed a rapid, visual, sensitive, and precise LAMP assay for the detection of *D. segeticola* identified from tea plants in Jiangsu, Yunnan, and Zhejiang Provinces in China based on sequence-characterized amplified regions. Two pairs of species-specific amplification primers (external primers F3 and B3 and internal primers FIP and BIP) targeting a specific sequence of *D. segeticola* were designed and screened. The specificity, sensitivity, and efficiency of the LAMP assay were determined, and disease diagnosis in the field was also performed. We demonstrated that the LAMP method can be used as a field-portable diagnostic assay with the advantages of simplicity, effectiveness, and visibility. The application of LAMP detection in field prediction and monitoring of tea leaf spot disease can lead to timely intervention in disease outbreaks and provide an important approach for developing sustainable strategies for the management of tea leaf spot.

## 2. Materials and Methods

### 2.1. Strains

A total of eight isolates of *Didymella segeticola* were collected from tea plant leaves, including YCW109 and YCW1111 from Jiangsu Province; YCW205 and YCW2007 from Yunnan Province; and YCW192, YCW1135, YCW1289, and YCW2184 from Zhejiang Province (Table 1). *Colletotrichum camelliae* LS_19, *D. coffeae-arabicae* YCW1972, *D. pomorum* YCW196, *D. sinensis* YCW1906 and YCW1950, and *Stagonosporopsis caricae* YCW1928 were used as controls in this study [52,53,54,55]. Six isolates of *D. segeticola* YCW192, YCW205, YCW1111, YCW1135, YCW1289, and YCW2184, and *C. camelliae* LS_19 were obtained from diseased leaves via single-spore isolation [52,54,55]. *Didymella coffeae-arabicae* YCW1972, *D. pomorum* YCW196, *D. sinensis* YCW1906 and YCW1950, and *S. caricae* YCW1928 were obtained by tissue isolation from healthy leaves. All the strains used in this study were identified based on morphological characteristics and multi-locus phylogenetic analysis [56]. The sequences of the ITS (the internal transcribed spacer region of the rDNA gene), LSU (partial large subunit nrDNA nucleotide sequences), *RPB2* (the RNA polymerase II second largest subunit gene), and *TUB2* (partial gene regions of β-tubulin) loci used for identification of the isolates can be obtained from GenBank^®^ (https://www.ncbi.nlm.nih.gov/genbank/, accessed on 20 May 2023) with accession numbers as listed in Table 1.

### 2.2. Culture Conditions and DNA Extraction

Isolates were cultured on potato dextrose agar (PDA) at 28 °C in the dark for 7 days. Aerial mycelia of each isolate were scraped from the plates and then subjected to DNA extraction. Genomic DNA was extracted from the collected mycelia using a Genomic DNA Purification Kit (Product number: B518259-0100, Sangon Biotechnology (Shanghai) Co., Ltd., Shanghai, China). DNA concentrations were determined spectrophotometrically using 260-nm absorbance with an ultra-micro ultraviolet-visible spectrophotometer ND-100C (MIULAB, Hangzhou, China) [49]. All DNA samples dissolved in sterile ddH_2_O were stored in DNase-free and sterile 1.5 mL centrifuge tubes at −20 °C.

### 2.3. LAMP Primer Design

The genome sequences of *D. segeticola* (GenBank accession number: GCA_004522025.1) and the dominant pathogenic fungi in tea plants (*C. camelliae*: GenBank accession number GCA_018853505.1; *C. fructicola*: GenBank accession number GCA_025558505.1; and *Pseudopestalotiopsis camelliae-sinensis*: unpublished) were obtained from the National Center for Biotechnology Information (NCBI) (http://www.ncbi.nlm.nih.gov, accessed on 27 September 2022) with accession numbers or from the assembly of the Illumina HiSeq PE150 platform (Beijing Novogene Bioinformatics Technology Co., Ltd., Beijing, China). BLAST analysis was used to explore DNA sequences which were exclusively present in the *D. segeticola* genome [57]. After repeated experiments, a species-specific DNA sequence (NCBI accession number: OR987684), which was predicted to encode a Cys2His2-zinc-finger (C2H2-ZNF) protein, was selected as the target sequence. Primer Explorer V5 (online web service, http://primerexplorer.jp/e/, accessed on 1 June 2023) was used for the design of the LAMP primers. The four LAMP primers used are listed in Table 2 and included two external primers (F3 and B3) and two internal primers (FIP and BIP). The primers used were synthesized by SUNYA Biotechnology Co., Ltd. (Hangzhou, China). The specificity and sensitivity of the LAMP primers were tested. For the specificity test, the DNA concentration was uniformly diluted to 10 ng/µL. To evaluate the sensitivity of the LAMP assays, the DNA extracted from three isolates of *D. segeticola* (YCW109, YCW1135, and YCW1289) was serially diluted 10-fold (from 10 ng/µL to 10 ag/µL) as a template for the experiment.

### 2.4. Optimization of LAMP Reaction Conditions

The LAMP reactions were performed in a 25 μL reaction mixture consisting of 2.5 µL of 10 × ThermoPol Buffer (Somersworth, NH, USA), 1.5 µL of 100 mM MgSO_4_, 3.5 µL of 10 mM dNTP mixture, 4 µL of 10 mM external primers (F3 and B3), 0.5 µL of 10 mM internal primers (FIP and BIP), 1 µL of *Bst* DNA Polymerase, Large Fragment (Nanjing Vazyme Biotech Co., Ltd., Nanjing, China), and 1 µL of DNA template, adjusted to a final volume of 25 μL with sterilized distillation-distillation H_2_O (ddH_2_O). To determine the optimal temperature and time of the LAMP reactions, the LAMP mixtures were incubated for 60 min at 55, 58, 60, 62, 65, 68, and 70 °C, and at 65 °C in a water bath for 15, 30, 45, 60, 75, and 90 min [34,35,37]. The reactions were terminated by heat inactivation at 95 °C for 2 min. Each product was confirmed by 2.0% agarose gel electrophoresis and photographed under a UV transilluminator. In addition, after the reactions, the LAMP products were directly observed by the unaided eye after the addition of 0.2 µL of SYBR Green I dye (10,000 × concentrate in DMSO, Coolaber, Beijing, China). With a positive reaction, the mixture was yellow-green in color, whereas with a negative reaction, the color of the mixture remained orange. The experiments were repeated three times.

### 2.5. Detection of D. segeticola in Tea Plant Leaves

Healthy leaves were collected from 5-year-old tea plants of *Camellia sinensis* cv. *Longjing43* (LJ43), cv. *Zhongcha102* (ZC102), cv. *Zhongcha108* (ZC108), and cv. *Zhongcha302* (ZC302). Then, the leaves were surface-sterilized with 75% alcohol and washed with sterilized ddH_2_O twice. Tea plant leaves were air dried and subsequently inoculated with mycelial discs of *D. segeticola* isolates via the wound inoculation method [58]. A 5-mm mycelial disc from *D. segeticola* was placed on the wound on both sides of the main vein (left and right sides) and cultured for 48 h at 25 °C during the day and 20 °C at night, with cycles of 14 h of light and 10 h of darkness and a relative humidity of 80% [58]. Leaves of LJ43 inoculated with sterilized ddH_2_O were treated as the negative control. Each treatment with three biological replicates was repeated three times. After 3 days, genomic DNA was extracted from diseased leaves using a Genomic DNA Purification Kit (Sangon Biotechnology (Shanghai) Co., Ltd., Shanghai, China) according to the manufacturer’s protocol. The same method was used to extract genomic DNA from healthy leaves as the negative control. The LAMP assay was used to detect the presence or absence of *D. segeticola* in tea plant leaves.

### 2.6. Detection of D. segeticola in Leaves from Fields

Healthy and diseased leaves from 18 germplasms of tea plants, named 2018-M89, 2018-M90, 2018-M95, 2018-M86, 2018-M100, 2021-WZ-7, AJ15, AJ16, AJ17, AJ18, 2021-20M, 2021-38M, 2021-19M, 2021-45M, 2021-2-2-M, 2021-01-M, 2021-2-6-M, and 2021-3-11-M, were collected from the tea garden of the Tea Research Institute, Chinese Academy of Agricultural Sciences. Healthy leaves were collected on 15 June 2023, and diseased leaves were collected on 7 August 2023. Genomic DNA was extracted from the leaves using the same method as above, and then subjected to LAMP assays. Diseased leaves of LJ43 inoculated with the *D. segeticola* strain YCW2184 in the laboratory were treated as the positive control. At least three replicates of each treatment were repeated at least two times.

## 3. Results

### 3.1. Specificity of the LAMP Assay

**Figure 1:**
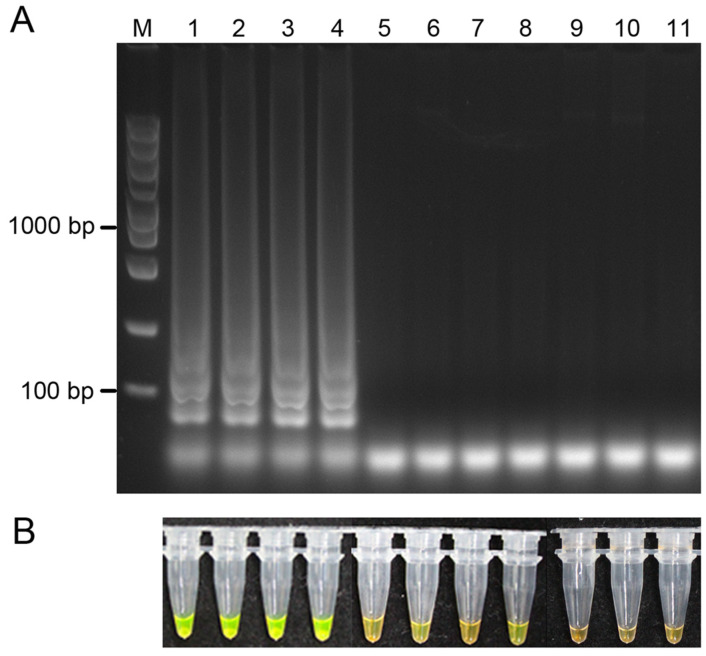
Specificity of the loop-mediated isothermal DNA amplification (LAMP) assay for detecting of *Didymella segeticola*. (**A**) Agarose gel electrophoresis of LAMP products of *D. segeticola* and other fungi. M, DL5000 DNA marker; Lane 1, *D. segeticola* YCW109; Lane 2, *D. segeticola* YCW1289; Lane 3, *D. segeticola* YCW1135; Lane 4, *D. segeticola* YCW205; Lane 5, *D. coffeae-arabicae* YCW1972; Lane 6, *D. pomorum* YCW196; Lane 7, *D. sinensis* YCW1906; Lane 8, *D. sinensis* YCW1950; Lane 9, *Stagonosporopsis caricae* YCW1928; Lane 10, *Colletotrichum camelliae* LS_19; Lane 11, no-template (ddH_2_O) control. (**B**) Color changes in LAMP products following the addition of SYBR Green I.

We first determined the specificity of LAMP assays with genomic DNA from *Didymella segeticola* and other fungi, *Colletotrichum camelliae*, *D. coffeae-arabicae*, *D. pomorum*, *D. sinensis*, and *Stagonosporopsis caricae*, which were isolated from tea plant leaves. Only samples from *D. segeticola*, which were isolated from tea plants in different areas, exhibited a ladder-like pattern in a 2% agarose gel after the reaction (Figure 1A). After the addition of SYBR Green I, the reaction products containing *D. segeticola* became yellowish-green (Figure 1B). In contrast, samples extracted from other fungi showed negative reactions. The results suggested that the LAMP assay can specifically detect *D. segeticola*.

### 3.2. Optimization of the LAMP Reaction Conditions

**Figure 2:**
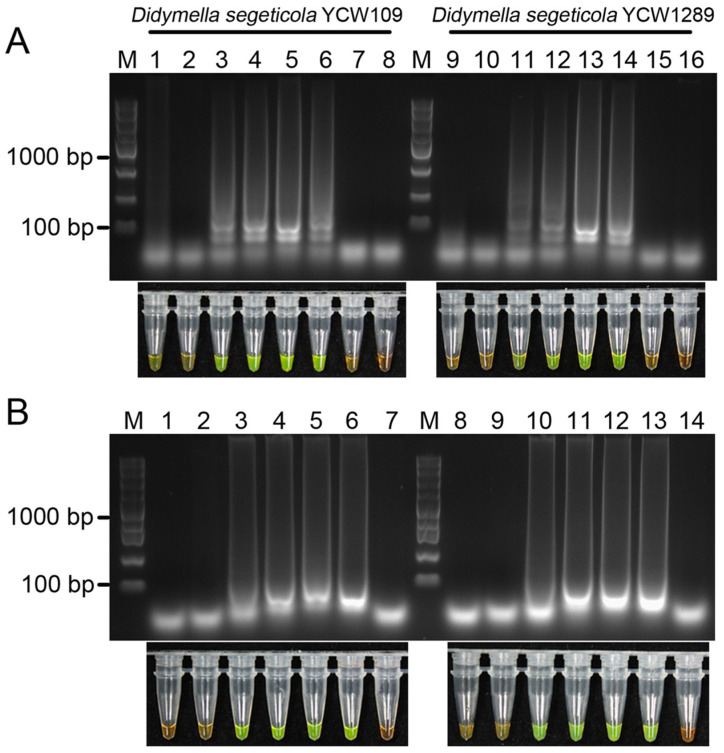
Optimization of the LAMP reaction conditions. (**A**) Reaction temperature screening. Lanes 1 and 9, 55 °C; Lanes 2 and 10, 58 °C; Lanes 3 and 11, 60 °C; Lanes 4 and 12, 62 °C; Lanes 5 and 13, 65 °C; Lanes 6 and 14, 68 °C; Lanes 7 and 15, 70 °C; Lanes 8 and 16, no-template (ddH_2_O) control. (**B**) Reaction time screening. Lanes 1 and 8, 15 min; Lanes 2 and 9, 30 min; Lanes 3 and 10, 45 min; Lanes 4 and 11, 60 min; Lanes 5 and 12, 75 min; Lanes 6 and 13, 90 min; Lanes 7 and 14, no-template (ddH_2_O) control. M, DL5000 DNA marker.

To optimize the LAMP reaction conditions, we carried out repeated assays using the genomic DNA of two *D. segeticola* isolates, YCW109 and YCW1289, as templates to determine the appropriate temperature and reaction time. A series of temperatures (55, 58, 60, 62, 65, 68, 70 °C) and durations (15, 30, 45, 60, 75, 90 min) were used for the LAMP assays according to previous methods. Ladder-like DNA fragments and discernible color changes were clearly observed in samples from both *D. segeticola* isolates heated at 60 °C to 68 °C for 60 min (Figure 2A). Similarly, positive reactions were clearly observed after 45–90 min of incubation at 65 °C. In addition, the ladder-like band was most clear at 65 °C and became more obvious after 60 min (Figure 2B). No ladder-like banding patterns with color changes were observed in the ddH_2_O control or other conditions. Thus, the LAMP assay applied for detecting *D. segeticola* was determined under the optimal conditions of 65 °C for 60 min.

### 3.3. Sensitivity of the LAMP Assays

**Figure 3:**
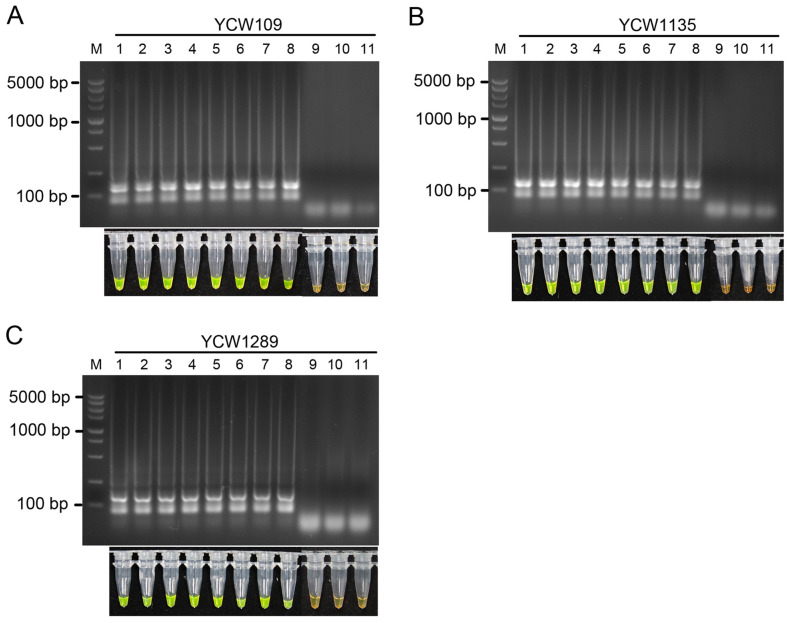
Sensitivity of the LAMP assay. Agarose gel electrophoresis and color changes of LAMP products with serially diluted genomic DNA (from 10 ng/µL to 10 ag/µL) from *D. segeticola* isolates YCW109 (**A**), YCW1135 (**B**), and YCW1289 (**C**) as templates. The detection limit was 1 fg/µL. The sensitivity of the assay was determined by the addition of SYBR Green I. The reactions were positive and presented a yellowish-green color. Lane 1, 10 ng/µL; Lane 2, 1 ng/µL; Lane 3, 100 pg/µL; Lane 4, 10 pg/µL; Lane 5, 1 pg/µL; Lane 6, 100 fg/µL; Lane 7, 10 fg/µL. Lane 8, 1 fg/µL; Lane 9, 100 ag/µL; Lane 10, 10 ag/µL; Lane 11, no-template (ddH_2_O) control. M, DL5000 DNA marker.

The sensitivity of the LAMP assay was accessed via amplification with 10-fold serial dilutions (from 10 ng/µL to 10 ag/µL) of genomic DNA extracted from three isolates of *D. segeticola* YCW109, YCW1135, and YCW1289 as templates. After reacting at 65 °C for 60 min, agarose gel electrophoresis clearly revealed ladder-like patterns at a minimal concentration of 1 fg/μL genomic DNA from the three isolates of *D. segeticola* (Figure 3A). After adding SYBR Green I to the reaction products, the products became yellowish-green (Figure 3B). No ladder-like banding patterns with color changes were observed in the ddH_2_O control or at the concentration of 100 ag/μL and 10 ag/μL genomic DNA from the three isolates. The minimum concentration of *D. segeticola* DNA detected for the LAMP assay was 1 fg/µL.

### 3.4. Using the LAMP Assay to Detect D. segeticola in Inoculated Tea Leaves

**Figure 4:**
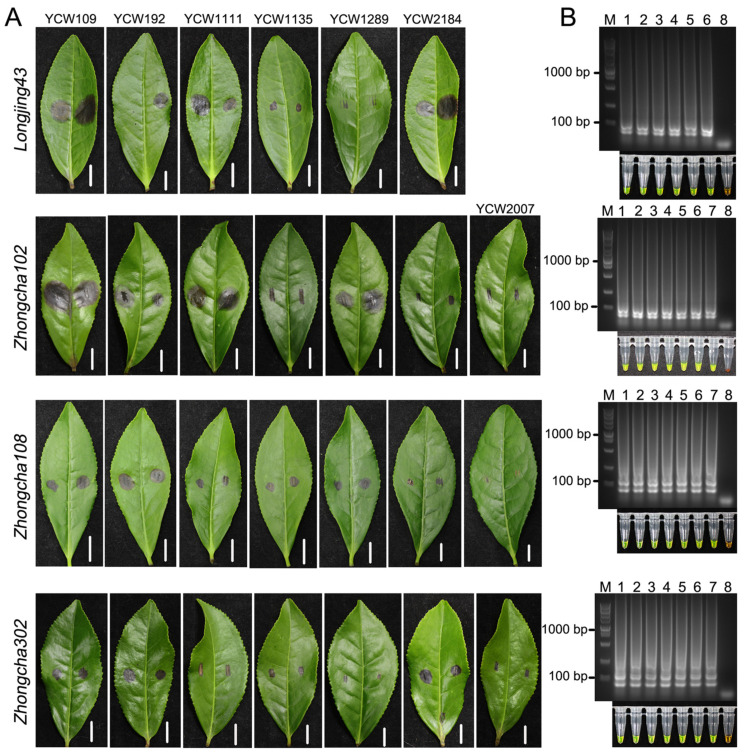
LAMP assay for detecting *D. segeticola* in inoculated tea leaves of LJ43, ZC102, ZC108, and ZC302. (**A**) Symptoms on leaves from different cultivars of tea plant (*Camellia sinensis*) 3 days after inoculation with *D. segeticola* isolates. (**B**) Agarose gel electrophoresis and color changes showing the LAMP results. The LAMP assays were performed using the genomic DNA from diseased leaves, which were inoculated with *D. segeticola* isolates, as templates. Lane 1: YCW109; Lane 2: YCW192; Lane 3: YCW1111; Lane 4: YCW1135; Lane 5: YCW1289; Lane 6: YCW2184; Lane 7: YCW2007; Lane 8: negative control (ddH_2_O). M, DL5000 DNA marker.

After determining the specificity, reaction conditions, and sensitivity of the LAMP assay, we subsequently performed the assay to detect *D. segeticola* in tea leaves. First, we inoculated the healthy tea leaves of LJ43, ZC102, ZC108, and ZC302 with *D. segeticola* isolates, respectively, via the wound-inoculation method (Figure 4A). After inoculation for 48 h, necrotic lesions were observed on mostly detached tea leaves. Then, total genomic DNA was extracted from diseased leaves and tested by a LAMP assay and evaluated by agarose gel electrophoresis and color changes with the addition of SYBR Green I. Although the aggressiveness of the isolates of *D. segeticola* varied, the reactions of all the inoculated samples were positive (Figure 4B). These results indicated that the LAMP assay can detect *D. segeticola* on the leaves of different tea plant cultivars.

### 3.5. Using the LAMP Assay to Detect D. segeticola in the Field

**Figure 5:**
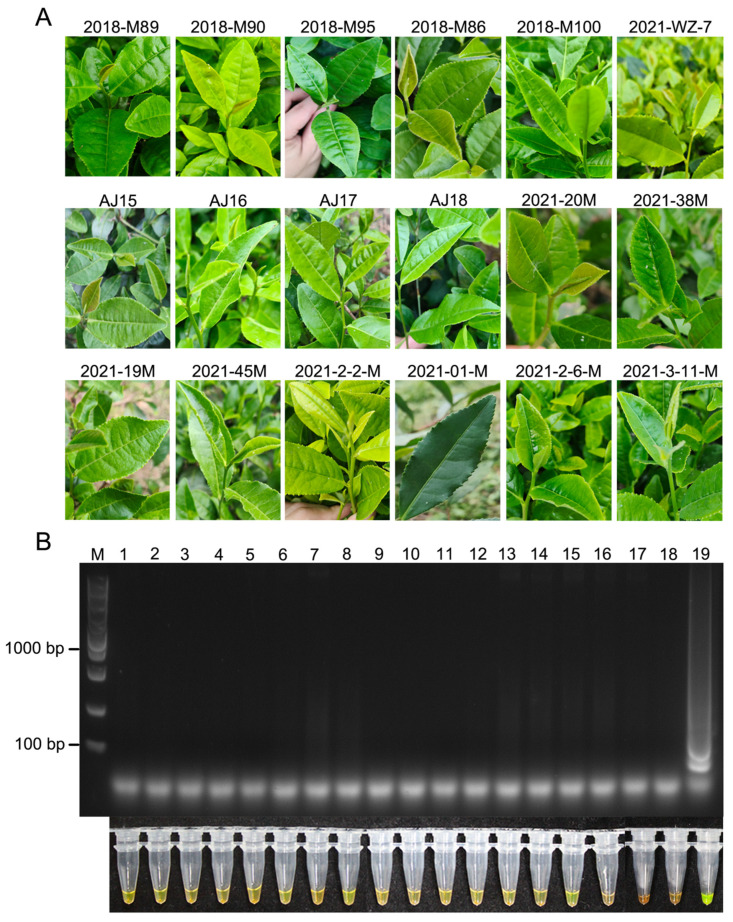
Detection of *D. segeticola* in healthy leaves of 18 germplasms of tea plants in the field. (**A**) Healthy leaves of different tea germplasms. (**B**) Agarose gel electrophoresis and color changes showing the LAMP results. Lane 1: 2018-M89; Lane 2: 2018-M90; Lane 3: 2018-M95; Lane 4: 2018-M86; Lane 5: 2018-M100; Lane 6: 2021-WZ-7; Lane 7: AJ15; Lane 8: AJ16; Lane 9: AJ17; Lane 10: AJ18; Lane 11: 2021-20M; Lane 12: 2021-38M; Lane 13: 2021-19M; Lane 14: 2021-45M; Lane 15: 2021-2-2-M; Lane 16: 2021-01-M; Lane 17: 2021-2-6-M; Lane 18: 2021-3-11-M; Lane 19: positive control (DNA extracted from diseased leaves of LJ43 inoculated with *D. segeticola* strain YCW2184). M, DL5000 DNA marker.

**Figure 6:**
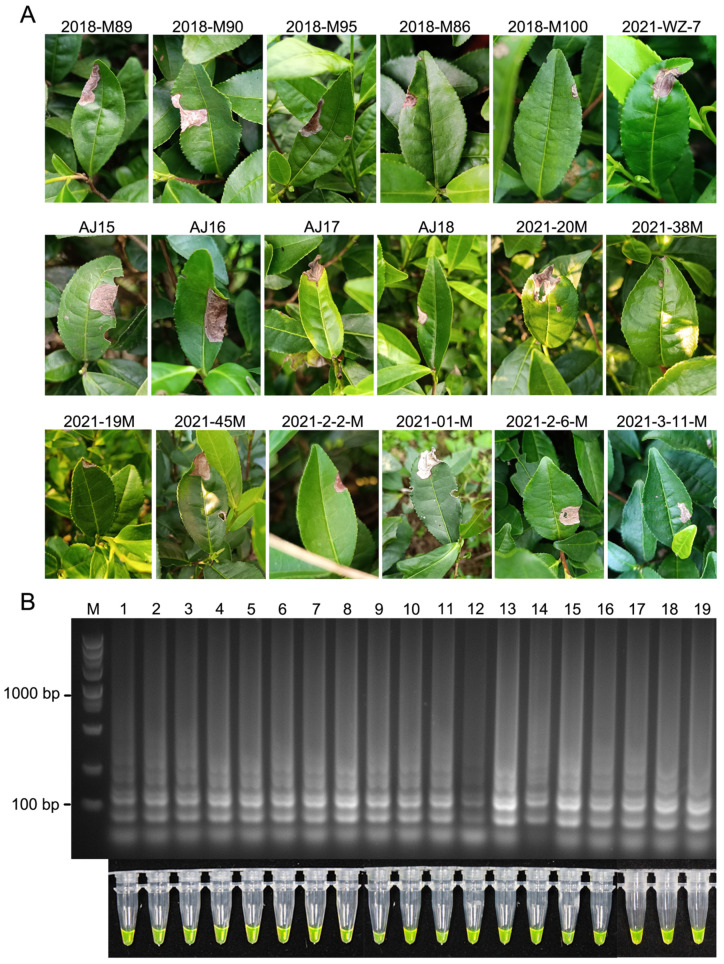
Detection of *D. segeticola* in diseased leaves of 18 germplasms of tea plants in the field. (**A**) Diseased leaves of different tea germplasms. (**B**) Agarose gel electrophoresis and color changes showing the LAMP results. Lane 1: 2018-M89; Lane 2: 2018-M90; Lane 3: 2018-M95; Lane 4: 2018-M86; Lane 5: 2018-M100; Lane 6: 2021-WZ-7; Lane 7: AJ15; Lane 8: AJ16; Lane 9: AJ17; Lane 10: AJ18; Lane 11: 2021-20M; Lane 12: 2021-38M; Lane 13: 2021-19M; Lane 14: 2021-45M; Lane 15: 2021-2-2-M; Lane 16: 2021-01-M; Lane 17: 2021-2-6-M; Lane 18: 2021-3-11-M; Lane 19: positive control (DNA extracted from diseased leaves of LJ43 inoculated with *D. segeticola* strain YCW2184). M, DL5000 DNA marker.

To further validate the usefulness of the LAMP assay, we used it to detect *D. segeticola* in healthy or diseased leaves of 18 germplasms of tea plants collected from the tea garden at the Tea Research Institute, Zhejiang Province. Total genomic DNA extracted from healthy or diseased tea leaves of the cultivars was used as the template for the LAMP assay. Health leaf samples tested negative for *D. segeticola* by the LAMP assay (Figure 5). Leaf samples with necrotic lesions from 18 germplasms of tea plants tested positive for *D. segeticola* in the LAMP assay (Figure 6). Therefore, the LAMP assay can be deployed in the field to rapidly detect *D. segeticola* in infected leaves.

## 4. Discussion

*Didymella segeticola* was first reported to cause leaf spot on the Tibetan thistle [59], and Zhao et al. (2018) reported that *D. segeticola* can cause wide occurrence of tea leaf spot in tea plantations in Guizhou Province [8]. Deng et al. (2023) reported that *D. segeticola* was the main pathogen causing leaf spot disease in commercial tea plantations in Guizhou and Sichuan Provinces [9]. Foliar disease on tea plants tends to occur during cold spell periods in tea plantations at higher altitudes or late spring, so it occurs mainly in southwestern China [8,58,60]. In other tea plantations, *D. segeticola* could also be isolated from infected tea leaves displaying leaf spots [61], implying that tea leaf spot caused by *D. segeticola* may spread gradually in the main tea cultivation provinces in China. However, as the most damaging and common foliar disease of tea plants, the epidemic of leaf spot has not been reported, and thus this disease can be difficult to control [9]. Thus, early diagnosis and rapid detection are highly important for limiting the spread of tea leaf spot in tea fields.

In this study, the LAMP assay was developed as a credible and sensitive diagnostic method for the detection of *D. segeticola* isolates from pure cultures as well as from infected samples in tea fields. To ensure the specificity of the LAMP assay for the detection of *D. segeticola*, we performed comparative genomic analysis using the genome sequences of the main pathogens identified from tea plants, including *Colletotrichum camelliae*, *C. fructicola*, *D. segeticola*, and *Pseudopestalotiopsis camelliae-sinensis* (we have assembled but cannot provide the genome information currently available). Many species-specific sequences were obtained and used to design the LAMP primers. After many attempts, we eventually identified a specific sequence as an appropriate and highly specific target for the design of LAMP primers. Based on BLASTN analysis, the sequence was predicted to encode a C2H2-ZNF protein. However, the biological functions of the protein have not been confirmed, so we submitted the sequence data to GenBank as a nucleotide sequence encoding a hypothetical protein (accession number: OR987684).

The LAMP technique involves loop insertions and strand displacement to perform entirely isothermal amplification, which requires at least four primers [22]. Two inner primers (FIP and BIP) invert sequences attached at their 5′ ends of F2/B2 regions and are used for strand displacement DNA synthesis [22,37,62]. Two outer primers (F3 and B3) anneal upstream of the inner primers, acting as binding sites for the *Bst* DNA polymerase, which has high displacement activity, and is used in the initial steps of the LAMP reactions [22,30,37,62]. In addition, two loop primers (forward loop F and backward loop B) can be used to achieve exponential amplification of LAMP and thus accelerate the reactions and improve the LAMP efficiency [37,62,63,64]. Studies have revealed that the time required for amplification with two loop primers is one-third to one-half of that required without a loop primer, and amplification can be achieved within 30 min [64]. In the process of primer screening, we also designed loop primers for LAMP detection in addition to inner and outer primers. However, the addition of loop primers results in difficulty in efficient amplification; therefore, we used four primers for the LAMP assays to ensure efficiency in this study. Similarly, numerous studies have chosen four primers to detect various plant pathogens via LAMP assays [35,37,65,66,67].

Based on the LAMP primer set designed for the amplification of target DNA sequences [68,69], we detected *D. segeticola* in different types of samples, including tea leaves infected with *D. segeticola*, healthy leaves, and suspected samples showing symptoms of leaf spots in the field. The LAMP reactions of samples from diseased tea leaves with suspected early symptoms were positive (Figure 4 and Figure 6), but the reactions of samples from healthy tea leaves were negative (Figure 5). The results confirmed that the LAMP assay could be used to directly detect *D. segeticola* in diseased samples from tea plants. Surprisingly, when tea leaf spots were severe, ladder-like DNA fragments and color changes were observed via the LAMP assay for the detection of *D. segeticola* in the young tea leaves without symptoms (Figure S1), which further suggested that the LAMP assay could be an efficient and sensitive method for the early diagnosis of leaf spots. In addition, the results of the sensitivity of the LAMP assays showed that reactions with 1 fg/µL *D. segeticola* genomic DNA as the template were still positive (Figure 3), indicating that the LAMP detection performed in this study was indeed highly sensitive.

The optimal conditions for the LAMP reaction for the detection of *D. segeticola* were at 65 °C for 60 min in a regular water bath, which provides isothermal conditions (Figure 2); this approach greatly reduced equipment requirements, and saved time and cost. However, LAMP has been used to develop a toolkit for detecting wheat blast caused by *Pyricularia oryzae*, which can provide results within 8 min as a quick pre-screening test [70,71]. Therefore, the LAMP assay in this study can be further optimized and improved in subsequent studies to more sensitively and efficiently detect *D. segeticola* from various samples in a shorter time. The genomic DNA used in the LAMP assay was extracted by an elaborate Genomic DNA Purification Kit at a price, which increases the procedure and cost of LAMP detection. Thus, the utilization of LAMP assays in fields can incorporate a quick and easy method for DNA extractions.

## 5. Conclusions

This is the first report describing a LAMP assay for the specific detection of *D. segeticola* in diseased tea leaves. Compared with other PCR detection methods, the developed LAMP assay, which has high operability, specificity, and sensitivity, is appropriate for the detection of *D. segeticola* and early diagnosis of tea leaf spot.

## Figures and Tables

**Table 1:** Isolates first used in this study and GenBank accession numbers of the generated sequences.

Species	Collecting Location	Tested Isolates	GenBank Accessions			
			ITS	*rpb2*	*tub2*	LSU
*Colletotrichum camelliae*	Lishui, Zhejiang, China	LS_19	MH463803	/	MH478602	/
*Didymella coffeae-arabicae*	Puer, Yunnan, China	YCW1972	OP647946	OP854293	/	/
*D. pomorum*	Yunnan, China	YCW196	OP647945	OP854292	OP854550	OP836938
*D. segeticola*	Yixing, Jiangsu, China	YCW109	OP647864	OP854211	OP854392	OP836867
	Lishui, Zhejiang, China	YCW192	OP647940	OP854287	OP854444	OP836877
	Yunnan, China	YCW205	OP647949	OP854296	OP854528	OP836907
	Wuxi, Jiangsu, China	YCW1111	OP647871	OP854218	OP854397	OP836869
	Hangzhou, Zhejiang, China	YCW1135	OP647884	OP854231	OP854507	OP836941
	Hangzhou, Zhejiang, China	YCW1289	OP647910	OP854257	OP854430	OP836875
	Puer, Yunnan, China	YCW2007	OP647948	OP854295	OP854512	OP836934
	Hangzhou, Zhejiang, China	YCW2184	OP647961	OP854308	/	OP836933
*D. sinensis*	Puer, Yunnan, China	YCW1906	OP647938	OP854285	OP854548	/
	Puer, Yunnan, China	YCW1950	OP647943	OP854290	OP854549	/
*Stagonosporopsis caricae*	Puer, Yunnan, China	YCW1928	OP648100	/	OP854594	OP837293

**Table 2:** Primers used for the LAMP assay to detect *D. segeticola*.

Primer	Sequence (5′-3′)
F3	CTTGGTCGAGCATAGAGCG
B3	CCTAGTCAGCACGGAACAG
FIP	CAGGTGGACGTGGCGTGTTGCACGACCGTTTTGCACAAC
BIP	AGCGATGCATGCACGAGCATAGGAAGAGAGATGACAGGGT

## Data Availability

The original contributions presented in the study are included in the article, further inquiries can be directed to the corresponding authors.

## Supplementary Materials

The following supporting information can be downloaded at: https://www.mdpi.com/article/10.3390/jof10070467/s1, Figure S1: LAMP assay for detecting Didymella segeticola in the young tea leaves without symptoms. Tea plants of LJ43 and ZC108 were respectively inoculated with the *D. segeticola* strain YCW2184. After about 20 days, the apical buds, first leaves and second leaves without symptoms of inoculated LJ43 and ZC108 were collected and prepared for the genomic DNAs extraction. Agarose gel electrophoresis and color changes showing the LAMP results. Lane 1: apical buds of LJ43; Lane 2: first leaves of LJ43; Lane 3: second leaves of LJ43; Lane 4: apical buds of ZC108; Lane 5: first leaves of ZC108; Lane 6: second leaves of ZC108. M, DL5000 DNA marker.
