# Supplementary material for: A Loop-Mediated Isothermal Amplification Assay for the Rapid Detection of Didymella segeticola Causing Tea Leaf Spot
Source: J Fungi (Basel). 2024 Jul 3;10(7):467. doi: 10.3390/jof10070467 (PMC11278140; doi:10.3390/jof10070467)
Supplement: Supplementary file 1 [file jof-10-00467-s001.zip › jof-3065972-supplementary.pdf]

A Loop-Mediated Isothermal Amplification Assay for the Rapid Detection of *Didymella segeticola* Causing Tea Leaf Spot

Yiyi Tu [1,†], Yuchun Wang [1,2,†], Hong Jiang [1], Hengze Ren [1], Xinchao Wang [1,2,*] and Wuyun Lv [1,2,*]

[1] College of Tea Science and Tea Culture, Zhejiang A&F University, Hangzhou 311300, China; 2022614022068@stu.zafu.edu.cn (Y.T.); ycwang0201@zafu.edu.cn (Y.W.); hulkww@zafu.edu.cn (H.J.); renhengze@zafu.edu.cn (H.R.)
[2] Tea Research Institute, Chinese Academy of Agricultural Sciences/National Center for Tea Improvement/Key Laboratory of Biology, Genetics and breeding of Special Economic Animals and Plants, Ministry of Agriculture and Rural Affairs, Hangzhou 310008, China
* Correspondence: wangxinchao@caas.cn (X.W.); lvwy@zafu.edu.cn (W.L.)
† These authors contributed equally to this work.

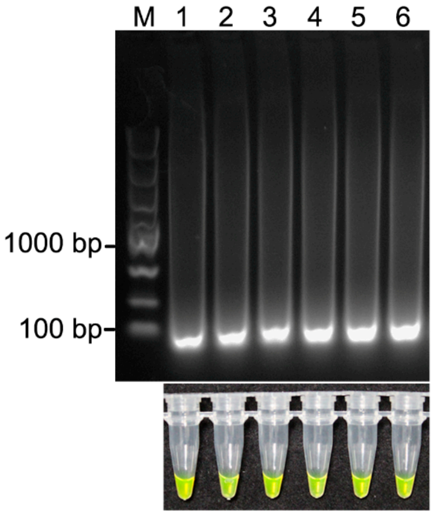

Figure S1. LAMP assay for detecting *Didymella segeticola* in the young tea leaves without symptoms. Tea plants of LJ43 and ZC108 were respectively inoculated with the *D. segeticola* strain YCW2184. After about 20 days, the apical buds, first leaves and second leaves without symptoms of inoculated LJ43 and ZC108 were collected and prepared for the genomic DNAs extraction. Agarose gel electrophoresis and color changes showing the LAMP results. Lane 1: apical buds of LJ43; Lane 2: first leaves of LJ43; Lane 3: second leaves of LJ43; Lane 4: apical buds of ZC108; Lane 5: first leaves of ZC108; Lane 6: second leaves of ZC108. M, DL5000 DNA marker.
